# A national retrospective study of the association between serious operational problems and COVID-19 specific intensive care mortality risk

**DOI:** 10.1371/journal.pone.0255377

**Published:** 2021-07-29

**Authors:** Harrison Wilde, John M. Dennis, Andrew P. McGovern, Sebastian J. Vollmer, Bilal A. Mateen

**Affiliations:** 1 Department of Statistics, University of Warwick, Coventry, United Kingdom; 2 Institute of Biomedical & Clinical Science, University of Exeter Medical School, Exeter, United Kingdom; 3 Diabetes and Endocrinology, Royal Devon and Exeter NHS Foundation Trust, Exeter, United Kingdom; 4 The Alan Turing Institute, London, United Kingdom; 5 Institute of Health Informatics, University College London, London, United Kingdom; BronxCare Health System, Affiliated with Icahn School of Medicine at Mount Sinai, NY, USA, UNITED STATES

## Abstract

**Objectives:**

To describe the relationship between reported serious operational problems (SOPs), and mortality for patients with COVID-19 admitted to intensive care units (ICUs).

**Design:**

English national retrospective cohort study.

**Setting:**

89 English hospital trusts (i.e. small groups of hospitals functioning as single operational units).

**Patients:**

All adults with COVID-19 admitted to ICU between 2nd April and 1st December, 2020 (n = 6,737).

**Interventions:**

N/A

**Main outcomes and measures:**

Hospital trusts routinely submit declarations of whether they have experienced ‘serious operational problems’ in the last 24 hours (e.g. due to staffing issues, adverse weather conditions, etc.). Bayesian hierarchical models were used to estimate the association between in-hospital mortality (binary outcome) and: 1) an indicator for whether a SOP occurred on the date of a patient’s admission, and; 2) the proportion of the days in a patient’s stay that had a SOP occur within their trust. These models were adjusted for individual demographic characteristics (age, sex, ethnicity), and recorded comorbidities.

**Results:**

Serious operational problems (SOPs) were common; reported in 47 trusts (52.8%) and were present for 2,701 (of 21,716; 12.4%) trust days. Overall mortality was 37.7% (2,539 deaths). Admission during a period of SOPs was associated with a substantially increased mortality; adjusted odds ratio (OR) 1.34 (95% posterior credible interval (PCI): 1.07 to 1.68). Mortality was also associated with the proportion of a patient’s admission duration that had concurrent SOPs; OR 1.47 (95% PCI: 1.10 to 1.96) for mortality where SOPs were present for 100% compared to 0% of the stay.

**Conclusion and relevance:**

Serious operational problems at the trust-level are associated with a significant increase in mortality in patients with COVID-19 admitted to critical care. The link isn’t necessarily causal, but this observation justifies further research to determine if a binary indicator might be a valid prognostic marker for deteriorating quality of care.

## Introduction

The emergence of the SARS-Cov-2 pathogen [1], and the new more transmissible variants [2], has resulted in large numbers of people, requiring hospital admission, often to high-acuity critical care settings [3]. In the UK for example, some hospitals increased their intensive care unit capacity by over 200% at the peak of the first wave of the COVID-19 pandemic to address the increased need [4]. Despite these re-deployed resources, and even in combination with the introduction of non-pharmacological interventions to limit disease transmission [5], many UK hospitals far exceeded the nationally-defined threshold of 85% for safe operating capacity [4]. However, the concerns about the operational strain on health systems extends beyond the more superficial capability to admit new patients, as there is also a well-established association between operational strain and individual-level patient outcomes, including those in critical care settings [6].

Evidence on the effect of operational strain on COVID-19 patient outcomes has begun to accumulate; for example, operating above the ‘safe occupancy threshold’ was associated with increased risk of COVID-19 mortality in a national cohort study of English intensive care units [7]. Similar results have been reported for the use of surge capacity (i.e. any beds that are made available above and beyond the baseline capacity of a critical care unit) in the USA and Europe [8, 9]. However, there are several limitations to using the methods employed by the aforementioned studies for defining operational strain. Firstly, the thresholds evaluated (most often for bed occupancy as a proxy for strain) reflect those set at the national level, meaning they do not reflect local or hospital-level nuances and context, and so are unlikely to provide accurate capture of local-level operational strain that could impact quality of care. Secondly, occupancy-based definitions do not capture staff absence levels. Previous research has suggested an impact of nursing and medical consultant staff absence on patient mortality risk in intensive care units [10]. Importantly, staff absence rates were raised 3-fold from the baseline of 4% at the peak of the first wave [11]. The potential impact of absence levels and other non-occupancy related operational strain on COVID-19 patient outcomes has not previously been evaluated, and may be of particular importance to understand drivers of the reported temporal improvements in in-hospital COVID-19 mortality over the course of the pandemic [12].

A potential solution to assessing the impact of local operational strain on outcomes is the generic ‘serious operational problems’ declaration (binary Yes/No flag) that English hospitals report as part of their daily situation reports (SitRep) to the national regulators [13]. These declarations are meant to reflect local-context defined operational issues in the preceding 24 hours [14], including those that do not rise to the standard of a regulator defined ‘serious incident’ (e.g. exceeding specific bed occupancy thresholds) [15]. In this study, we sought to describe the pattern of these generic ‘serious operational problem’ declarations over the course of the COVID-19 pandemic in England, and investigate the association between these declarations and COVID-19 specific mortality risk in intensive care units across England.

## Materials and methods

### Study population

**Fig 1:**
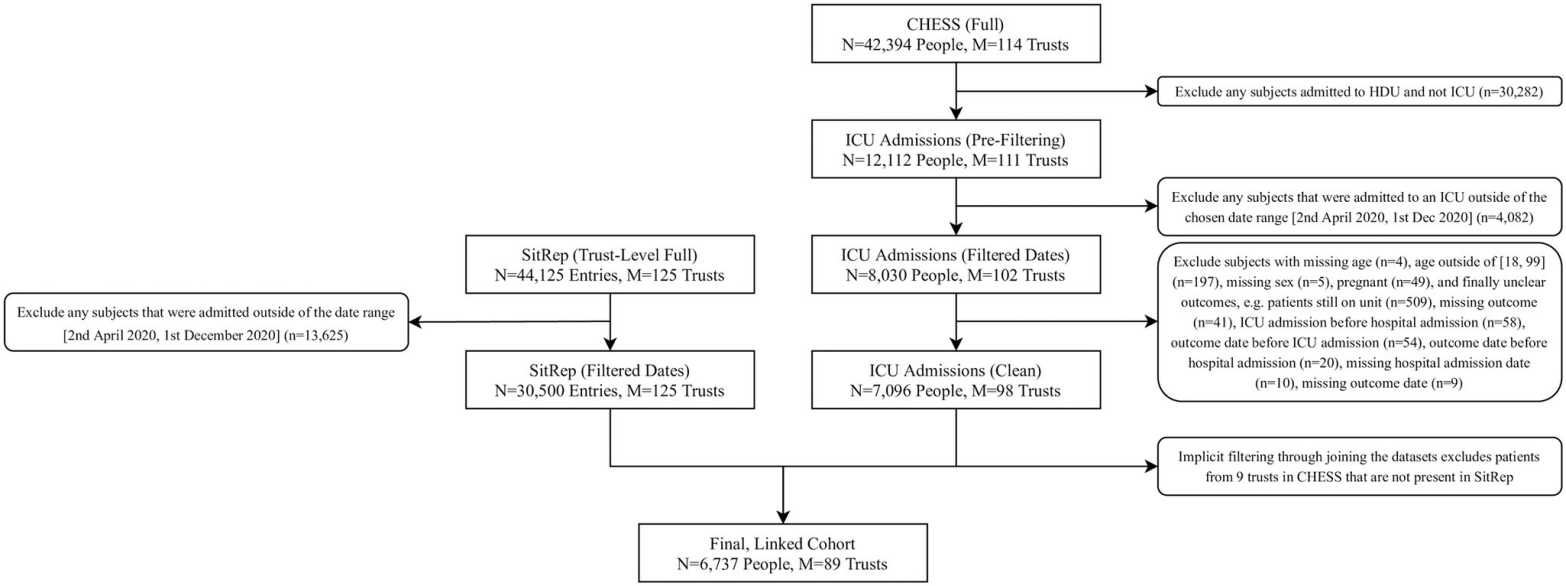
Flowchart detailing application of inclusion/exclusion criteria. A flowchart illustrating the filtering of raw data to reach the study population, through application of the inclusion and exclusion criteria to the two datasets (CHESS and SitRep), prior to joining for each individual based on date of admission and the trust to which the individual was admitted.

Eligible individuals were identified through a recorded admission in the COVID-19 Hospitalisation in England Surveillance System (CHESS), which captures confirmed or presumed COVID-19 related intensive care unit (ICU) admissions during the pandemic (diagnostic criteria described below) [16]. Submission of data to CHESS was made mandatory for hospitals in England. The earliest date for which all relevant data is available was the 2^nd^ of April 2020. All individuals admitted after this date, but before the end of the 1^st^ of December (i.e. the first day of de-escalation of the non-pharmacological interventions following the second ‘lockdown’ in England) were included. Individuals were eligible (see Fig 1 for a summary) if:

1. they were between the age of 18–99 years;
2. not known to be pregnant;
3. had gender recorded, and;
4. had consistent timestamps for admission and outcome.

#### Diagnostic criteria

CHESS comprises both presumed and confirmed cases of COVID-19. Presumed cases are defined as individuals with clinically diagnosed COVID-19 (whom never had a positive confirmatory diagnostic test) during the study period. However, 100% of people included here had some form of positive test. A positive test could include reverse transcriptase polymerase chain reaction (PCR) of any respiratory system specimen, an antibody, or antigen-based test.

#### Recorded clinical features

For each individual we extracted the following information: administrative data (admitting hospital trust (trusts being groups of hospitals that function as part of a single operational unit), date of admission, first segment of postcode (called the ‘outward code’)), demographics (age, sex, and ethnicity), and comorbidities coded as binary indicator variables with missingness equated to absence (chronic respiratory disease including asthma; obesity; diabetes; chronic heart disease; hypertension; immunosuppression due to disease or treatment; chronic neurological disease; chronic renal disease) [16]; the validity of this approach to coding CHESS comorbidities has been previously evaluated [17]. Although data on severity of illness at admission is not available in CHESS, a recent report from the national intensive care audit (ICNARC) showed no significant variation in mean severity score (APACHE-II) of those being admitted over the course of 2020 [18].

#### Recorded operational data

Administrative data was accessed from ‘SitReps’; the daily situation reports hospital trusts routinely submit on bed occupancy as well as including a declaration (binary Yes/No flag) of whether they have experienced ‘serious operational problems’ in the last 24 hours. These submissions are signed off by each trust chief executive at 11am each day, based on the data that reflects the trusts position at 8am on that day. Conceptually these ‘serious operational problems’ flags represent an operational issue (e.g. staffing issues, adverse weather conditions, a healthcare service in their network suffering from its own operational problems resulting in network-side effects, or even a wild animal disrupting clinical care provision [19]), which has led to a perceived or objective deterioration in service provision. In the UK, there can be strict definitions for the latter, but only when they rise to the level of a ‘serious incident’ as defined by the statutory regulator [20], which usually requires direct discernible harm to an individual (or a near miss). However, there are often circumstances where discernible harm is not detected, but local administrators are still able to identify an operational problem that compromised service provision. The guidance to trust administrators is to “use their judgement on what, other than an issue which leads to a ‘serious incident’ as defined by the national framework, constitutes a serious operational problem” [14]. As such, there is no singular list of reasons for which a hospital might declare that they experienced a serious operational problem, rather these are based on local standards and are highly context dependent, and thus can be likened to self-reported symptom elicitation from a patient. Moreover, the exact reason (in narrative form) underlying the serious operational problem is not collected by the regulator as far as we are aware.

Alongside extracting whether a trust declared a ‘serious operational problems’, occupancy of general and acute beds, as well as mechanical ventilator occupancy (as a marker of intensive care unit strain) were extracted. Occupancy was defined as the proportion of surge capacity occupied on each calendar day. The full cleaning and preparation of the SitRep data is described in detail elsewhere [4], however, for this analysis an additional step was necessary as there are three dates where no data is available: 14^th^ May, 24^th^ May and 24^th^ November. To address this issue, a naïve imputation method was applied where the occupancy information for the preceding 24-hour period were used to forward-fill the dates in question.

#### Outcome

All-cause in-hospital mortality with follow-up until death, discharge, or transfer, where both latter conditions are considered absorbing states indicating survival (the data did not distinguish whether a discharge was part of a palliative care plan or not, and thus this is a limitation of the dataset). Individuals still on the ward on the date of final observation (i.e. 22^nd^ December), were excluded from the primary analysis, but included in sensitivity analyses. Otherwise, no date restriction was applied to the in-hospital follow-up ensure that patients with long stays were not automatically assumed to survive, which might bias our results.

### Statistical analysis

Descriptive summaries were generated as follows: medians with interquartile ranges for continuous variables and counts and proportions for categorical data.

#### Bayesian model specification

Bayesian hierarchical models (with default, flat priors on categorical features and student’s t priors on smooths and group level effects) were used to estimate, as odds ratios, the association between mortality and: 1) an indicator for whether a serious operational incident occurred on the date of a patient’s admission, and; 2) the proportion of the days in a patient’s stay that had a serious operational incident occurring within their relevant trust.

Adjustments were made for the following coefficients: *Age* (in years), *calendar week of ICU Admission*, *Sex*, indicators for the presence of the following chronic co-morbidities: *Respiratory Disease(s)* (including Asthma), *Immunosuppressive Disease*, *Renal Disease*, *Hypertension*, *Heart Disease*, *Liver Disease*, *Neurological Disease* and *Diabetes*. *Week of ICU Admission* was treated linearly following investigation with a spline that showed no evidence of non-linearity. *Age* was treated as a continuous variable and was re-parameterised using a cubic spline with 4 knots to investigate and subsequently represent non-linearity in the effect. *Sex* and the various comorbidity indicators are dichotomous. *Chronic Respiratory Disease(s)* represents the union of two initially separate covariates in the data: *Respiratory Disease* and *Asthma*, setting instances where both are either missing or no to be negative; otherwise positive. Alongside the aforementioned population level effects, the primary models included some group level effects: *Ethnicity* was included with intercept coefficient for each of 7 groups: White, Asian Subcontinent, Asian (Other), Black, Mixed, Other and Missing; *Obesity* was included with intercept coefficients for each of 3 groups: Obese, Non-Obese and Missing; and *Trust* was included with intercept coefficients for all 90 trusts.

The model parameter space was sampled using Hamiltonian Monte Carlo with 3 chains of 3,000 iterations each, using the Stan statistical programming language [21]. The target proposal acceptance probability was modified from the default value to 0.95 to improve convergence with the hierarchical shrinkage prior. All models discussed had fewer than 1 in 1000 divergent transitions and R-hat diagnostics of 1.00. The minimum bulk effective sample size was 2000 for group coefficients and 2700 for population coefficients, and 3000 for smooth ones. All modelling was carried out using the BRMS package [22], in R [23].

#### Sensitivity analysis

To support the primary analysis, multiple sensitivities were formulated and undertaken to ensure the results seen were robust to changes in model structure and various assumptions. Specifically, these sensitivities covered: filtering for different degrees of missingness at trust-level by removing all trusts from the modelling data with a total of 25% / 50% / 75% of all comorbidity information missing; adjustment for the time taken in days between a patient’s admission to hospital and their escalation to ICU; adjustment for occupancy on the calendar date of admission, and; adjustment for patient-level deprivation index as a proxy for socioeconomic status—derived through taking the weighted average of all super-output areas that fall within outward code available for every individual. Finally, sensitivity analyses were carried to ensure that the lack of final outcomes for some patients did not bias the modelling results. The primary model was fitted only on those patients that had a definite final outcome and associated date within the specified study cohort date range. As such, we fit the same primary models but on different datasets: including those patients that were said to be still on the unit at the time of the extract (n = 509), assuming they all survived; and another including those patients with clear final outcomes, but no final outcome date provided (n = 9).

### Ethics & governance

The study was approved by the Warwick Biosciences Research Ethics Committee (BSREC 120/19-20-V1.1) and sponsorship is being provided by University of Warwick (SOC.28/19-20). The raw data was collected by Public Health England (PHE) as part of their statutory responsibilities, which allows them to process patient confidential data without explicit patient consent, and using the additional statutory powers granted during the COVID-19 pandemic were empowered to share the data with specific academic groups for the purposes of research (i.e., the COPI notices). In this study we utilised a de-identified version of this dataset, with the assent of the BSREC, who recognised that the aforementioned statutory justifications were sufficient to proceed without informed consent from individual patients.

#### Patient and public involvement

No patients were involved in the design, interpretation of the results, or dissemination of this study.

## Results

6,737 patients with COVID-19 were admitted to intensive care units within the included trusts (Fig 1), which resulted in 122,008 patient-days observed. 2,539 deaths were recorded (37.7%), equating to a mortality rate of 20.8 per 1000 patient-days. Baseline characteristics are summarised in Table 1. 47/90 trusts (52.8%) reported at least one day of serious operational problems, with a total of 2,701 trust-days (of 21,716; 12.4%) associated with serious operational problems. Table 2 summarises the characteristics of trusts that experienced serious operational problems and those that didn’t.

**Table 1:** Characteristics of the study cohort stratified by whether there was a serious operational problem occurring on their date of admission, in their admitting trust.

	Incident on Date of Admission		Overall
	Yes	No	
	(n = 1,085)	(n = 5,652)	(n = 6,737)
**Age in Years**			
Median [IQR]	61 [51, 69]	60 [51, 68]	60 [51, 68]
**Time in Days to ICU from Hospital Admission**			
Median [IQR]	1 [0, 4]	1 [0, 3]	1 [0, 3]
**Age Group**			
18–24	11 (1.0)	53 (0.9)	64 (0.9)
25–34	30 (2.8)	214 (3.8)	244 (3.6)
35–44	98 (9.0)	468 (8.3)	566 (8.4)
45–54	217 (20.0)	1,153 (20.4)	1,370 (20.3)
55–64	302 (27.8)	1,769 (31.3)	2,071 (30.7)
65–74	304 (28.0)	1,343 (23.8)	1,647 (24.4)
75–84	109 (10.0)	570 (10.1)	679 (10.1)
85+	14 (1.3)	82 (1.5)	96 (1.4)
**Sex**			
Female	356 (32.8)	1,770 (31.3)	2,126 (31.6)
Male	729 (67.2)	3,882 (68.7)	4,611 (68.4)
**Ethnicity**			
White	714 (65.8)	3,245 (57.4)	3,959 (58.8)
Asian Subcontinent	90 (8.3)	597 (10.6)	687 (10.2)
Asian (Other)	41 (3.8)	369 (6.5)	410 (6.1)
Black	46 (4.2)	389 (6.9)	435 (6.5)
Mixed	14 (1.3)	107 (1.9)	121 (1.8)
Other	29 (2.7)	341 (6.0)	370 (5.5)
Missing	151 (13.9)	604 (10.7)	755 (11.2)
**Obesity**			
Obese	382 (35.2)	2,313 (40.9)	2,695 (40.0)
Non-Obese	428 (39.4)	1,939 (34.3)	2,367 (35.1)
Missing	275 (25.3)	1,400 (24.8)	1,675 (24.9)
**Comorbidity**			
Diabetes	261 (24.1)	1,482 (26.2)	1,743 (25.9)
Chronic Respiratory Disease(s)	247 (22.8)	1,186 (21.0)	1,433 (21.3)
Chronic Heart Disease	117 (10.8)	680 (12.0)	797 (11.8)
Chronic Renal Disease	79 (7.3)	461 (8.2)	540 (8.0)
Chronic Neurological Disease	45 (4.1)	292 (5.2)	337 (5.0)
Chronic Liver Disease	23 (2.1)	158 (2.8)	181 (2.7)
Immunosuppressive Disease	20 (1.8)	206 (3.6)	226 (3.4)
Hypertension	348 (32.1)	1,965 (34.8)	2,313 (34.3)
**Proportion of Days in Stay Where an Incident Occurred**			
Median [IQR]	1.0 [0.8, 1.0]	0 [0, 0]	0 [0, 0]
**Weighted IMD**			
Median [IQR]	4.7 [3.4, 6.6]	4.6 [3.2, 6.5]	4.6 [3.2, 6.5]
**Mortality**			
Crude (Unadjusted, Absolute)	433 (39.9)	2106 (37.3)	2539 (37.7)

**Table 2:** Characteristics of trusts stratified by whether they reported a serious operational problem during the study period.

	SOP During Study Period		Overall
	No	Yes	
	(n = 42)	(n = 47)	(n = 89)
**Bed Availability**			
Baseline	22 [13, 50]	18 [11, 27]	19 [13, 36]
Peak	48 [31, 98]	47 [32, 65]	47 [31, 89]
**Bed Occupancy**			
Median	0.50 [0.40, 0.63]	0.50 [0.40, 0.62]	0.50 [0.40, 0.62]
Mean	0.51 [0.42, 0.62]	0.53 [0.43, 0.60]	0.52 [0.43, 0.60]
Min	0.17 [0.11, 0.27]	0.13 [0.09, 0.20]	0.14 [0.10, 0.23]
Max	0.95 [0.88, 1.00]	1.00 [0.86, 1.00]	0.95 [0.86, 1.00]
**Weighted IMD**			
Median	4.63 [3.96, 5.92]	4.76 [4.18, 6.23]	4.71 [4.14, 6.09]
Mean	5.03 [4.18, 5.69]	5.10 [4.30, 6.11]	5.08 [4.26, 5.84]
**Mortality**			
	0.36 [0.26, 0.43]	0.40 [0.29, 0.48]	0.37 [0.28, 0.45]
**Count in Region**			
East of England	3	5	8
London	11	6	17
Midlands	4	9	13
North East and Yorkshire	5	9	14
North West	6	5	11
South East	6	7	13
South West	7	6	13

### Deconstructing the serious operational problem declaration

**Fig 2:**
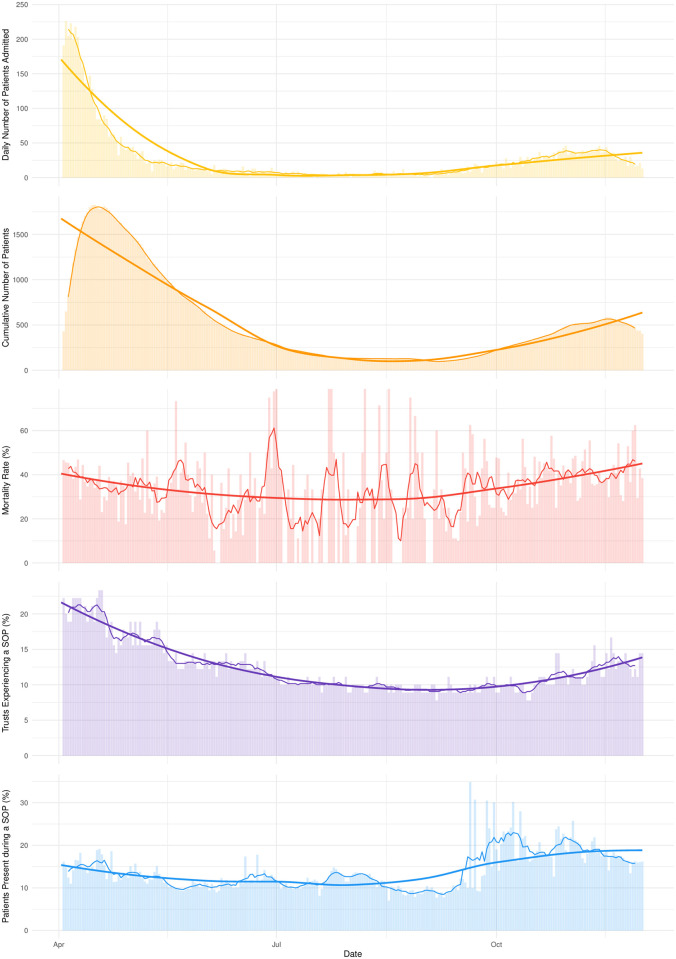
Histograms of: 1) the daily number of admissions recorded in CHESS (Yellow); 2) the cumulative number of patients present on each day (Orange); 3) the mortality rate over the study period (Red); 4) the percentage of trusts experiencing a SOP on each day (Purple); 5) and the percentage of all patients in CHESS that were admitted to trust experiencing a SOP on each day (Blue), respectively from top to bottom. Each histogram is based on the 7-day rolling mean and loess smoothed curves. As such, there is a lagged peak in the cumulative number of patients present on each day (Orange), where in fact the real peak of the first wave in the UK was around the 22^nd^ of April 2020. Moreover, the substantial variance seen in mortality rate throughout the summer months is due the 7-day rolling average across intermittently low numbers of daily admissions recorded in CHESS during this period. Interestingly, the distribution of serious operational problems (Purple) implies that despite the fewer trusts experiencing SOPs at the end of the study, more patients were affect (Blue), possibly due to larger size trusts experiencing SOPs during the winter months.

Fig 2 illustrates the distribution of SOPs at patient and trust levels alongside the COVID-19 epidemic curve. Notably, the administration-reported SOPs are not solely a reflection of the incidents that might be reported due to exceeding nationally defined occupancy thresholds (i.e. 85%), as 92.8% of serious operational problems (i.e. 2,506 of the 2,701 trust days) occurred on dates when reported occupancy was less than the national 85% threshold.

### Serious operational problems on the date of admission

**Fig 3:**
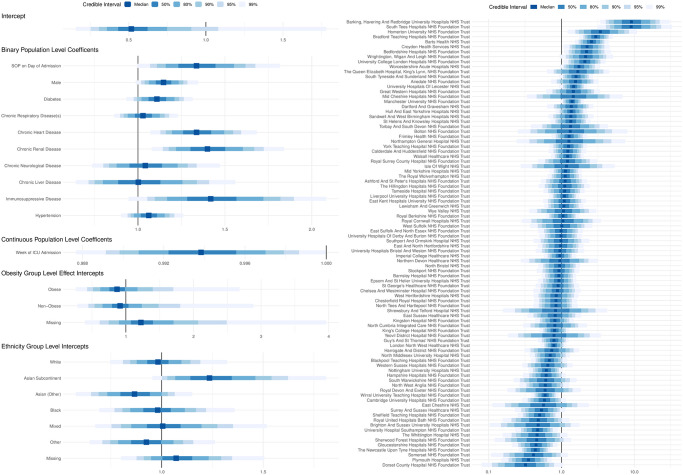
Forest plot of all co-variates in the final model where a binary flag indicating that a serious operational problem occurred on the date of admission. (Left) A forest plot of all the effect estimates and marginal posterior densities for each covariate in the final model in which a serious operational problem occurred on the date of admission was captured using a binary flag (Yes/No). (Right) The marginal posterior densities (on a log odds scale) for the random effect for each trust (i.e. small group of hospitals that function as a single operational unit), in the aforementioned model.

The unadjusted odds ratio (OR) for mortality based on whether a patient was admitted during a period of serious operational problems was 1.12 (95% posterior credible interval (PCI): 0.98 to 1.28, 90% PCI: 1.00 to 1.25). Following adjustment for patient-level factors (full model specification shown in Fig 3), the OR was 1.34 (95% CI: 1.07 to 1.68, 90% CI: 1.11 to 1.62). The results of the sensitivity analyses, detailed in Table 3, illustrate that the associations are not explained by data missingness, occupancy on date of admission, or modelling structure and assumptions.

**Table 3:** Marginal posterior densities for the primary effects of interest, under the various sensitivity analyses that were carried out.

	Posterior Credible Intervals				Median
	95%		90%		
	Lower	Upper	Lower	Upper	
**Primary**					
SOP on Day of Admission	1.07	1.68	1.11	1.62	1.34
Proportion of Stay with an SOP	1.10	1.96	1.16	1.87	1.47
**With Time to ICU in Days (Continuous)**					
SOP on Day of Admission	1.07	1.69	1.11	1.62	1.34
Proportion of Stay with an SOP	1.12	1.96	1.16	1.87	1.47
**With Occupancy of Beds Supporting Mechanical Ventilation (Linear Proportional)**					
SOP on Day of Admission	1.09	1.70	1.13	1.64	1.36
Proportion of Stay with an SOP	1.10	1.95	1.15	1.85	1.46
**With Weighted Index of Multiple Deprivation (Decile)**					
SOP on Day of Admission	1.07	1.65	1.10	1.60	1.33
Proportion of Stay with an SOP	1.10	1.95	1.15	1.85	1.45
**75% Missingness Threshold**					
SOP on Day of Admission	1.09	1.70	1.13	1.64	1.36
Proportion of Stay with an SOP	1.12	1.97	1.18	1.88	1.48
**50% Missingness Threshold**					
SOP on Day of Admission	1.04	1.64	1.08	1.59	1.31
Proportion of Stay with an SOP	1.07	1.92	1.12	1.83	1.44
**25% Missingness Threshold**					
SOP on Day of Admission	1.07	1.75	1.11	1.66	1.36
Proportion of Stay with an SOP	1.05	1.97	1.11	1.87	1.43
**Data Including Patients that were Still on the Unit as of 22nd December (Add. n = 509)**					
SOP on Day of Admission	1.06	1.64	1.11	1.59	1.32
Proportion of Stay with an SOP	1.02	1.74	1.06	1.66	1.32
**Data Including Patients that were Missing a Final Outcome Date (Add. n = 9)**					
SOP on Day of Admission	1.07	1.66	1.11	1.60	1.33
Proportion of Stay with an SOP	1.11	1.94	1.16	1.87	1.46

### Length of stay associated with serious operational problems

**Fig 4:**
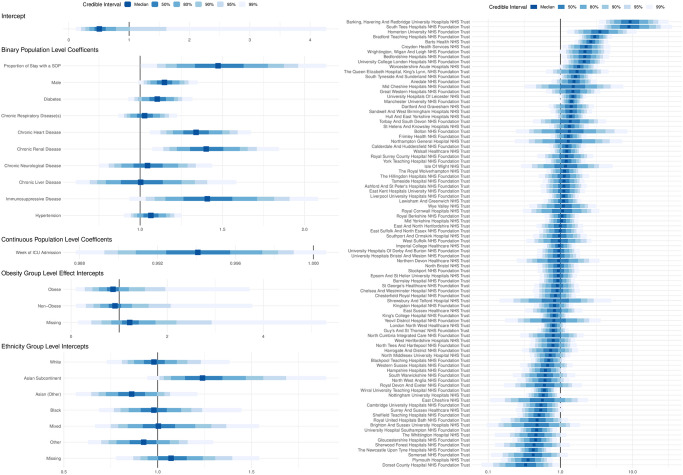
Forest plot of all co-variates in the final model in which the proportion of a patient’s stay during which the relevant trust was experiencing a serious operational problem was the exposure of interest. (Left) A forest plot of all the effect estimates and marginal posterior densities for each covariate in the final model in which the proportion of a patient’s stay during which the relevant trust was experiencing a serious operational problem was the exposure of interest. (Right) The marginal posterior densities (on a log odds scale) for the random effect for each trust (i.e. small group of hospitals that function as a single operational unit), in the aforementioned model.

The unadjusted OR for risk of mortality for the proportion of a patient’s admission duration that had serious operational problems (i.e. 100% stay compared to 0% of stay) was 1.14 (95% CI: 0.99 to 1.31, 90% CI: 1.01 to 1.28), whilst the fully adjusted OR for risk of mortality for the proportion of the admission duration with SOPs was 1.47 (95% CI: 1.10 to 1.96; 90% CI: 1.16 to 1.87, see Fig 4). Again, this finding was robust in all sensitivity analysis (Table 3).

## Discussion

Our study shows the declaration of serious operational issues by hospitals appears to be associated with a substantially increased critical care mortality for patients with COVID-19. The size of association is comparable to those observed for high-risk patient-level factors such as the presence of major co-morbidities, for example diabetes [17]. Importantly, we demonstrate that declaration of serious operational issues does not simply reflect hospital occupancy levels, as over 93% of declarations were reported on days when hospitals did not exceed nationally agreed upon occupancy standards and the association was not attenuated after adjustment for ventilated bed occupancy.

### In context of the literature

Our study is the first to evaluate the potential influence of hospital trust-level serious operational problems on critical care outcomes for patients with COVID-19. The findings are concordant with the previous studies that have evaluated occupancy levels (one type of serious operational problem) and shown that operating at extremes of critical care capacity is associated with worse COVID-19 outcomes [7–9]. The fact that a positive association was seen in our study when adjusting for occupancy (both on the date of admission and during admission) in sensitivity analysis provides clear evidence that considering patient occupancy levels alone is insufficient to provide a complete picture of operational strain in secondary care. Further work to disentangle the effects of specific serious operational problems, in particular staff absence levels, would be of considerable interest in datasets where such information is available.

### Strengths and limitations

Strengths of this study include the use of a national-level dataset (CHESS) which provided near complete capture of COVID-19 intensive care admissions in England in 2020. CHESS is a mandatory collection for hospitals in England, meaning ascertainment of mortality outcomes should be well recorded. Moreover, the use of a robust Bayesian framework allowed us to more accurately model the uncertainties implicit in the analysis, and reflect these in the reported parameter values, which are often lost by the maximum likelihood (or least squares) estimated methods are often employed for similar analyses.

A limitation of the CHESS dataset is the lacked of validated patient level clinical data, in particular comorbidity for which data completeness is variable at hospital trust level, and a lack of reliable information on patient acuity at admission. Our robust sensitivity analysis, showing consistent estimates of association when filtering for different degrees of missingness at trust-level, mitigates concern over potential bias from hospital trust level coding variation. Whilst previous analysis of UK national critical care during COVID-19 suggests differences in patient acuity at admission are not associated with mortality [18], further analysis incorporating patient-level severity information such as the APACHE-II score alongside hospital level operational data would be of considerable interest. A final limitation is that operational problems in this study were reported at the trust level, not specifically relating to the functioning of the intensive care unit, and thus there is a risk of an ecological bias which must be acknowledged.

### Implications for researchers and policy makers

Although limited by the fact that our results should not be interpreted causally, this study highlights the potential for using generic serious operational problem flags to capture hospital operational issues where more granular data capture is not appropriate or feasible. This is likely to be an important tool both for responding to the current COVID-19 pandemic, but also more generally for service evaluation and performance monitoring long after. Moreover, the marked association between trust-level operational problems and individual-level outcomes in critical care setting highlights the need for additional research to identify the specific causal factors that might be driving the association. Novel modelling methods are more than capable of handling unstructured natural language data, and might be able to identify modifiable factors around which to develop mitigation strategies to improve future patient care—as such, policy makers should consider prioritising the collection of free-text descriptions, and developing mechanisms to facilitate the sharing of this data with researchers alongside the generic flags upon which this study is based.

## Conclusions

Using a national dataset, our findings demonstrate that serious operational problems occurring at hospital trust level are associated with a substantial increase in mortality in patients with COVID-19 admitted to intensive care units. These generic serious operational issue flags potentially offer insight on local-level operational strain beyond that provided by assessment of bed occupancy levels which did not explain the associations we observed. However, there are also a number of other factors that may have contributed to the deterioration in quality of care which would not be captured in such a flag, from human error to organisational culture, and which we were not able to correct for. In essence, further research to disentangle the causal pathways between operational and organisational factors and mortality outcome, and prospective evaluation of the SOP binary indicator to determine if it is a valid prognostic marker (including outside of the pandemic context) for deteriorating quality of care, are both necessary.
